# Effects of Dietary 25-Hydroxycholecalciferol Alone or in Combination with Vitamin D_3_ on Growth Performance, Blood Vitamin D Status, Immune Response, Bone Integrity, and Antioxidant Capacity of Nursery Pigs

**DOI:** 10.3390/ani16050771

**Published:** 2026-03-01

**Authors:** Chan Ho Kwon, Eva S. Safaie, Jannell A. Torres, Zhaohui Yang, Xi Chen, Pengcheng Xue, Young Dal Jang

**Affiliations:** 1 Department of Animal and Dairy Science, University of Georgia, Athens, GA 30602, USA; 2 Nutribins LLC, Covina, CA 91723, USA

**Keywords:** 25-hydroxycholecalciferol, antioxidant status, bone integrity, growth performance, immunity, nursery pigs, vitamin D_3_

## Abstract

This study evaluated the effects of dietary 25-hydroxycholecalciferol alone or in combination with vitamin D_3_ (VD_3_) on growth performance, vitamin D status, immune parameters, bone integrity, and antioxidant capacity in nursery pigs. Sixty weanling piglets (5.63 ± 0.98 kg initial body weight) were assigned to three treatments for a 28 d feeding trial. The treatments were a basal diet supplemented with 2000 IU/kg VD_3_, a combination of 1000 IU/kg VD_3_ and 1000 IU/kg 25-hydroxycholecalciferol (25-OHD_3_), and 2000 IU/kg 25-OHD_3_. Pigs fed diets containing 25-OHD_3_ tended to have greater feed intake during d 14–28 (*p* = 0.06). Plasma 25-OHD_3_ concentrations were greater in pigs fed diets containing 25-OHD_3_ than in those fed VD_3_ alone on d 14 and 28 (*p* < 0.05), with greater values observed in pigs fed 2000 IU/kg 25-OHD_3_ than in those fed a combination of 1000 IU/kg VD_3_ and 1000 IU/kg 25-OHD_3_ on d 14 (*p* < 0.05). Pigs fed diets supplemented with 25-OHD_3_ had lower plasma malondialdehyde concentrations at d 28 (*p* < 0.05), while immune parameters and bone mineralization indicators were unaffected. In the broken-line analysis, the estimated plasma 25-OHD_3_ concentration for plasma malondialdehyde concentrations to reach the minimum level was 32.5 ng/mL (*p* < 0.05). In conclusion, supplementing 25-OHD_3_ in nursery diets improved blood vitamin D status and could help reduce oxidative stress during the late nursery period.

## Simple Summary

Vitamin D is commonly added to nursery pig diets, and 25-hydroxycholecalciferol (25-OHD_3_) is a form that is more bioavailable than vitamin D_3_ (VD_3_). In this study, we evaluated the effects of replacing VD_3_ with 25-OHD_3_ in diets for nursery pigs on growth, vitamin D status, bone integrity, and antioxidant capacity. Pigs fed diets containing 25-OHD_3_, either alone or combined with VD_3_, consumed more feed than those fed VD_3_ alone. In addition, higher circulating vitamin D levels and lower oxidative stress were observed in pigs fed diets supplemented with 25-OHD_3_, suggesting improved health status after weaning. However, immune measurements and bone integrity were not affected by the supplementation of 25-OHD_3_. Overall, supplementing 25-OHD_3_ in nursery diets enhanced vitamin D status and helped alleviate postweaning oxidative stress.

## 1. Introduction

Vitamin D plays essential roles in skeletal development, mineral homeostasis, immune function, and antioxidant defense in pigs [1,2,3,4]. Traditionally, vitamin D has been supplemented in swine diets in the form of vitamin D_3_ (VD_3_), whereas 25-hydroxycholecalciferol (25-OHD_3_) has been recently introduced and commercially adopted as an alternative vitamin D source [2]. Compared with VD_3_, 25-OHD_3_ is known to have higher bioavailability due to its ability to bypass hepatic conversion, greater absorption efficiency, and reduced metabolic degradation [5,6,7]. In previous studies, it has been reported that 25-OHD_3_ supplementation in nursery diets improved the postweaning growth rate, feed intake, and antioxidant defense system and reduced oxidative stress in pigs [7,8,9,10], indicating that 25-OHD_3_ supplementation could alleviate weaning stress in pigs. However, these studies used diets in which 25-OHD_3_ completely replaced VD_3_ [8] or added 25-OHD_3_ to control diets formulated to contain 1600–2000 IU/kg of VD_3_ [7,9,10], resulting in total vitamin D supplementation levels that exceeded typical industry standards (1600–2500 IU/kg) [11]. Thus, it is important to evaluate the efficacy of 25-OHD_3_ as a partial or complete replacement for VD_3_ while maintaining the total vitamin D equivalent level at 2000 IU/kg to avoid confounding effects of elevated total vitamin D levels and unnecessary over-supplementation in weaning pigs. In addition, 25-OHD_3_ supplementation in nursery diets has been reported to improve immune function and bone integrity under Ca- and P-deficient conditions [12,13], whereas studies using adequate Ca and P levels have reported limited or no effect on immunoglobulin (Ig) production or skeletal traits [14,15]. Thus, it is important to understand the physiological role of 25-OHD_3_ under nutritionally adequate conditions when used as a replacement for VD_3_ in nursery diets. Therefore, the objective of the present study was to evaluate the effects of dietary 25-OHD_3_, used to partially or completely replace VD_3_, at an equivalent total vitamin D level (2000 IU/kg) on growth performance, blood vitamin D status, antioxidant capacity, immune responses, and bone mineralization in weaned pigs fed diets containing normal Ca and P levels.

## 2. Materials and Methods

### 2.1. Animals, Experimental Design, and Housing

At weaning, a total of 60 newly weaned pigs (Camborough × PIC337 and [Camborough × Berkshire] × PIC337; 5.63 ± 0.98 kg initial body weight; weaned at 17.9 ± 1.59 d of age) were allotted to 1 of 3 dietary treatments in 5 replicates with 4 pigs (2 barrows and 2 gilts) per pen based on body weight, breed, sex, and littermate in a randomized complete block design for a 28 d feeding trial. The treatments were (1) VD: basal diet with 2000 IU/kg VD_3_ supplementation; (2) MIX: basal diet with 1000 IU/kg VD_3_ + 1000 IU/kg 25-OHD_3_ supplementation; and (3) 25OHD: basal diet with 2000 IU/kg 25-OHD_3_ supplementation. In this study, VD_3_ was partially (1000 IU/kg) or completely (2000 IU/kg) replaced with 25-OHD_3_ to evaluate its efficacy as an alternative source of vitamin D. All the pigs were housed in nursery pens (1.0 m × 2.0 m) with woven-wire flooring, fed ad libitum, and had free access to water in an environmentally controlled nursery facility at the University of Georgia Large Animal Research Unit. No creep feed was provided during the lactation period. The pigs were fed the same treatment diets with two diet phases, including d 0–14 postweaning (Phase 1), and d 14–28 postweaning (Phase 2). The 25-OHD_3_ product (SmartD) was obtained from Nutribins LLC (Covina, CA, USA).

### 2.2. Experimental Diets

All the pigs were fed corn–soybean meal-based diets in mash form that were formulated to meet or exceed nutrient requirement estimates of NRC [16] for 7–11 kg (Phase 1) and 11–25 kg (Phase 2) pigs (Table 1). The VD_3_ supplementation level (2000 IU/kg) in the VD diet was consistent with the average level commonly used in U.S. nursery pig diets as the control diet [11]. The 25OHD diet was supplemented with dietary 25-OHD_3_ at a level equivalent to 2000 IU VD_3_/kg. For the MIX diet, both VD_3_ and 25-OHD_3_ were supplemented at 1000 IU/kg each, providing a total VD level equivalent to 2000 IU VD_3_/kg. To minimize differences in the non-treatment components of the diets, a basal diet was first mixed with a VD_3_-free vitamin premix without the inclusion of corn starch. The basal diet was divided into 3 equal fractions. The treatment diets were mixed by adding a premix containing either VD_3_ or 25-OHD_3_ prepared by blending the respective vitamin D source with corn starch.

### 2.3. Data and Sample Collection

The pigs were individually weighed at the start of the trial (d 0), d 7, 14, 21, and 28 postweaning. The pen-based feed disappearance was measured when the pigs were weighed. The average daily gain (ADG), average daily feed intake (ADFI), and gain-to-feed (G:F) ratio were calculated. Blood samples (10 mL) were collected from eight pigs per treatment (2 pigs per pen from the first 4 replicates; 1 barrow and 1 gilt) selected based on average body weight in each pen on d 14 and 28 postweaning via jugular venipuncture in disposable vacutainer tubes containing the anticoagulant K_3_ EDTA (Becton Dickinson, Franklin, NJ, USA). Plasma samples were obtained by centrifugation at 2500× *g* for 30 min at 4 °C and stored at −80 °C until analysis. At d 28 postweaning, 6 pigs per treatment (3 barrows and 3 gilts) were selected based on average body weight from first 3 replicates, with 1 barrow and 1 gilt per pen to reduce potential variations from the pigs with small body size in the last 2 replicates. These pigs were euthanized by the captive bolt method to collect femur samples that were analyzed for bone mineral content (BMC) and bone mineral density (BMD) using dual energy X-ray absorptiometry (GE Healthcare, Chicago, IL, USA).

### 2.4. Chemical Analysis

The plasma 25-OHD_3_ concentrations were analyzed by Heartland Assays (Ames, IA, USA). The plasma IgG and IgA concentrations were determined using commercial ELISA kits (Bethyl Laboratories, Montgomery, TX, USA), and antioxidant parameters, including superoxide dismutase activity (SOD), total antioxidant capacity (T-AOC), and malondialdehyde (MDA) concentrations, were measured using colorimetric assay kits (Cayman Chemical Company, Ann Arbor, MI, USA), all following the manufacturer’s instructions, with absorbance measured using a spectrophotometer (Thermo Fisher Scientific, Waltham, MA, USA).

### 2.5. Statistical Analysis

All the data obtained in the current study were analyzed in accordance with a randomized complete block design using the PROC MIXED procedure of SAS (ver. 9.4; SAS Inst. Inc., Cary, NC, USA). A pen was used as an experimental unit for the analysis of growth performance data. An individual pig was used as an experimental unit for blood and bone analyses. The models included the treatment as a fixed effect and the replicate as a random effect for growth performance and the replicate within pen and the pen as a random effect for blood and bone parameters. A single degree of freedom contrast was performed to make a comparison between VD_3_ treatment vs. combined 25-OHD_3_ treatments (MIX and 25OHD treatments). The least square means were separated using the PDIFF option of SAS. PROC REG in SAS was used to analyze the relationships between plasma 25-OHD_3_ concentrations and antioxidant (T-AOC, SOD, and MDA), immunological (IgG and IgA), and bone mineralization parameters (BMD and BMC) within each sampling day. When significant quadratic correlations were observed, the linear broken-line regression analysis was performed using PROC NLIN to estimate the plasma 25-OHD_3_ concentrations to reach the minimum or maximum blood or bone parameters as described by Robbins et al. [17]. Statistical differences were established at *p* < 0.05 and tendencies were established at 0.05 ≤ *p* < 0.10.

## 3. Results and Discussion

### 3.1. Growth Performance

There were no significant differences observed in the overall growth performance among the dietary treatments. When the two 25-OHD_3_ treatments were combined, the pigs fed diets supplemented with 25-OHD_3_ tended to have greater ADFI in d 14–21 (*p* = 0.09), d 21–28 (*p* = 0.06), and d 14–28 (*p* = 0.06) and lower G:F ratio in d 21–28 (*p* = 0.08) postweaning than those fed diets supplemented with only VD_3_ (Table 2). The increasing ADFI trend observed in the current study aligns with our previous study [8], which reported that pigs fed a diet containing 25-OHD_3_ had an increased ADFI compared to those fed VD_3_ during late nursery period. One potential reason for the increased feed intake is the reduction in plasma MDA levels at d 28 postweaning, indicating that 25-OHD_3_ may help alleviate postweaning oxidative stress, which is associated with reduced feed intake and impaired growth in piglets [18]. Although it was not examined in the present study, Menendez et al. [19] reported that VD_3_ can directly suppress leptin secretion from adipose tissue in an in vitro study, and lower circulating leptin concentrations are generally associated with increased appetite. Therefore, the result of the current study suggests that dietary 25-OHD_3_ supplementation may enhance postweaning feed intake in pigs during the nursery period.

In the current study, although pigs fed 25-OHD_3_ supplemented diets tended to have greater postweaning feed intake during the late nursery period, ADG was not affected, resulting in a tendency toward a lower G:F ratio. Similarly, previous studies have reported that dietary 25-OHD_3_ supplementation had no effect in enhancing growth rate during the nursery period, although dietary 25-OHD_3_ supplementation levels were over 2000 IU/kg [9,10,20]. However, our previous study [8] reported increased feed intake and growth rate in the late nursery period by feeding pigs with diets supplemented with 25-OHD_3_ at 2000 IU/kg compared to VD_3_ supplementation at the same level. Although both studies observed increased feed intake with dietary 25-OHD_3_ supplementation, the current study did not show an increase in the growth rate during the late nursery period. Interestingly, the plasma 25-OHD_3_ concentrations at d 28 postweaning in the current study were greater than those observed in our previous study, despite the same supplementation level and source of 25-OHD_3_. The reason for the discrepancy in growth responses between the studies remains unclear; however, it may suggest that once an adequate vitamin D status is achieved, further increases in plasma 25-OHD_3_ concentrations may not elicit additional growth responses. Similarly, Witschi et al. [20] reported no difference in the growth rate between pigs supplemented with VD_3_ or 25-OHD_3_ at 2000 IU/kg, whereas pigs receiving a low level of VD_3_ (200 IU/kg) exhibited reduced growth performance. Therefore, the growth response to vitamin D supplementation may be more pronounced under inadequate vitamin D conditions, with limited additional benefits beyond a certain threshold of vitamin D status. In addition, greater feed intake alone in this study may not be sufficient to enhance growth rate, as increased feed intake in nursery pigs undergoing postweaning gastrointestinal development may not always proportionally translate into body weight gain [21]. Taken together, further studies are needed to clearly demonstrate the relationship between growth performance and blood vitamin D status in nursery pigs.

### 3.2. Plasma 25-OHD_3_ Concentrations

Pigs receiving dietary 25-OHD_3_ showed greater plasma 25-OHD_3_ concentrations than those fed VD_3_ at both d 14 and 28 postweaning (*p* < 0.05; Table 3), regardless of the levels. However, pigs in the 25OHD group exhibited 1.38- and 1.20-fold greater concentrations than those in the MIX group at these respective time points, with significantly greater values observed in the 25OHD group than the MIX group at d 14 postweaning. These findings align with previous studies showing that dietary 25-OHD_3_ supplementation increases plasma 25-OHD_3_ concentrations in a dose-dependent manner [8,20,22]. Moreover, the current results indicate that supplementation with 25-OHD_3_ alone is more effective at elevating blood vitamin D levels than VD_3_ alone or combined supplementation of VD_3_ and 25-OHD_3_ at the same VD level, which agrees with previous studies [8,9,20,22,23]. Interestingly, despite equivalent total dietary vitamin D inclusion (2000 IU/kg) across vitamin D sources, the pigs in the present study had greater plasma 25-OHD_3_ concentrations than those observed in our previous experiment [8]. This result suggests that circulating vitamin D status may be affected by multiple factors, including dietary vitamin D inclusion levels. One possible reason can be that the pigs in the current study had 10–13% greater overall feed intake compared to our previous study [8], resulting in increased 25-OHD_3_ intake, which may have contributed to increased plasma 25-OHD_3_ concentrations. In addition, Madson et al. [24] reported that diarrheic pigs could have lower vitamin D status due to vitamin D malabsorption, indicating that the health status of pigs might influence circulating 25-OHD_3_ levels. Although the underlying mechanism remains unclear, these findings indicate that multiple factors may influence plasma 25-OHD_3_ concentrations, although the source and level are the major factors in increasing circulating 25-OHD_3_ levels.

### 3.3. Immunological Responses

In the present study, dietary 25-OHD_3_ supplementation did not affect plasma IgG or IgA concentrations at d 14 or 28 postweaning (Table 4). Plasma IgG and IgA are key B-cell-derived indicators of humoral immune status in pigs and have been linked to enhanced immune function and resilience to weaning stress when their plasma concentrations are elevated [14]. Thus, the results of the current study indicate that dietary 25-OHD_3_ supplementation had no significant effects on systemic humoral immune status of the pigs in the nursery period. Consistent with the present findings, no differences in serum IgG concentrations were observed between pigs fed a basal diet containing 2500 IU/kg of VD_3_ and those fed the same diet supplemented with 2000 IU/kg of 25-OHD_3_ [25] when graded 25-OHD_3_ was supplemented from 0 to 4000 IU/kg [14] or when 25-OHD_3_ inclusion increased from 220 to 6220 IU/kg under porcine epidemic diarrhea virus challenge conditions [15]. Similarly, Zhou et al. [7] reported no differences in serum IgA concentrations between pigs fed a basal diet containing 2000 IU/kg of VD_3_ and a diet with an additional 2000 IU/kg of VD_3_ or 25-OHD_3_. In contrast, Zhang et al. [12] reported that dietary 25-OHD_3_ supplementation increased serum IgG concentrations under calcium- and phosphorus-deficient conditions, suggesting that dietary 25-OHD_3_ supplementation may support humoral immunity primarily under nutritionally compromised conditions. These findings indicate that dietary 25-OHD_3_ may not have an influence on humoral immunity under nutritionally adequate conditions.

### 3.4. Bone Mineralization Parameters

In the current study, no significant differences in BMD or BMC were observed among the dietary treatments (Table 5), indicating that dietary 25-OHD_3_ supplementation did not affect bone mineralization during the nursery period. Consistent with the present findings, Becker et al. [10] reported no effects of supplementing 2000 IU/kg of 25-OHD_3_ on bone density or bone ash content in nursery pigs fed a basal diet providing 1653 IU/kg of VD_3_, and von Rosenberg et al. [9] similarly observed no differences in bone ash, calcium, or phosphorus contents when comparing VD_3_ (2000 IU/kg) with increasing levels of 25-OHD_3_ supplementation ranging from 2000 to 20,000 IU/kg. However, Zhang et al. [12] demonstrated that supplementation with 2000 IU/kg of 25-OHD_3_ in low-calcium and -phosphorus diets increased serum calcium concentrations, as well as bone biochemical markers, including bone-specific alkaline phosphatase and osteocalcin, indicating enhanced bone metabolism under mineral-deficient conditions. These findings suggest that dietary 25-OHD_3_ supplementation may not enhance bone mineralization under nutritionally adequate conditions.

### 3.5. Plasma Antioxidant Parameters and Correlation Analysis

**Figure 1:**
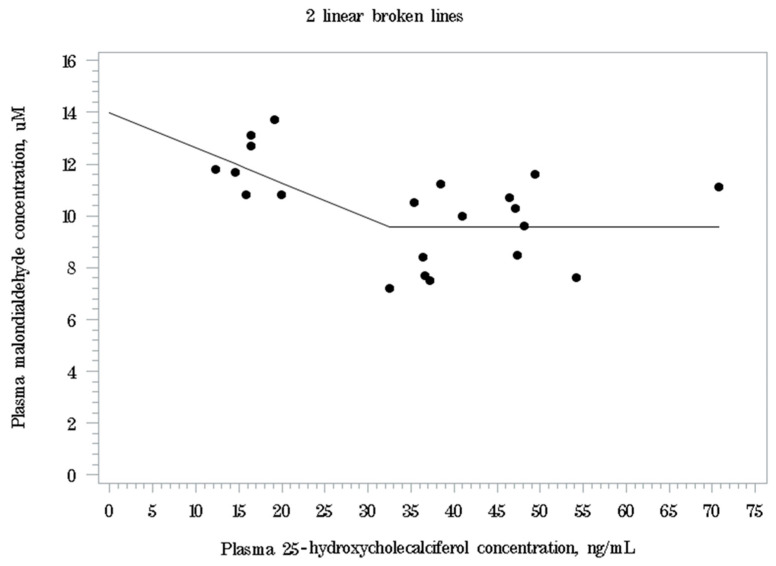
Broken-line analysis of plasma malondialdehyde (MDA) level by plasma 25-hydroxycholecalciferol (25-OHD_3_) concentrations at d 28 postweaning. The breakpoint was estimated at 32.5 ng/mL (*p* < 0.05). The estimated regression equation was plasma MDA levels, µM = 9.57 + 0.136 × (32.5 − 25-OHD_3_) if plasma 25-OHD_3_ < 32.5 ng/mL and plasma MDA level, µM = 9.57 if plasma 25-OHD_3_ ≥ 32.5 ng/mL.

In the plasma antioxidant parameters, there were no significant differences among dietary treatments in SOD activity and T-AOC (Table 6). The plasma MDA levels of pigs decreased by dietary 25-OHD_3_ supplementation (MIX or 25OHD treatment) compared with the VD_3_ treatment (*p* < 0.05) at d 28 postweaning. These results indicate that increasing 25-OHD_3_ supplementation level could reduce oxidative stress of weaning pigs in late nursery period, which agrees with previous studies [7,8]. A quadratic correlation was observed only between the plasma 25-OHD_3_ and MDA levels at d 28 postweaning (quadratic: *p* < 0.05, R^2^ = 0.486) among the antioxidant status parameters. Based on linear broken-line analysis, the estimated plasma 25-OHD_3_ concentration for the plasma MDA level to reach the minimum level was 32.5 ng/mL (*p* < 0.05; Figure 1). The estimated regression equation for the plasma MDA levels (µM) is MDA = 9.57 + 0.136 × (32.5 − plasma 25-OHD_3_) when the plasma 25-OHD_3_ level is less than 32.5 ng/mL. When the plasma 25-OHD_3_ level is greater than or equal to 32.5 ng/mL, the plasma MDA level reaches its minimum at 9.57 µM. These results indicate that increasing plasma 25-OHD_3_ concentrations are associated with a reduction in oxidative stress up to a threshold of 32.5 ng/mL, beyond which no further decrease in plasma MDA levels is observed. This result agrees with our previous study [8] reporting that plasma MDA levels reached the minimum values when the plasma 25-OHD_3_ levels reached 23.7 ng/mL. Interestingly, this estimated breakpoint of plasma 25-OHD_3_ levels was greater in the current study compared to our previous study (32.5 vs. 23.7 ng/mL) [8], although both studies used the same source of dietary 25-OHD_3_ at levels up to 2000 IU/kg. The reason for this discrepancy remains unclear; however, differences in the plasma 25-OHD_3_ levels between the current and previous studies, despite pigs being fed the same VD_3_ or 25-OHD_3_ supplementation levels, may partially explain these results. Although there are differences in the estimated breakpoint values, both studies consistently indicate that supplementation with VD_3_ alone, even at an inclusion level of 2000 IU/kg, could not achieve plasma 25-OHD_3_ concentrations corresponding to the lowest plasma MDA levels. Although further studies are necessary to precisely define the plasma 25-OHD_3_ threshold required to minimize oxidative stress in weaning pigs, dietary supplementation with 25-OHD_3_, either alone or in combination with VD_3_, is necessary to achieve plasma 25-OHD_3_ concentrations associated with minimal oxidative stress.

## 4. Conclusions

At an equivalent total vitamin D level (2000 IU/kg), partial or complete replacement of VD_3_ with 25-OHD_3_ resulted in greater plasma 25-OHD_3_ concentrations compared with VD_3_ alone. Although the growth rate, blood immunoglobin levels, and bone mineralization were not affected, supplementing 25-OHD_3_ in nursery diets could reduce oxidative stress, as indicated by lower plasma MDA levels. The plasma MDA level was correlated with plasma 25-OHD_3_ concentrations and minimized when the plasma 25-OHD_3_ concentrations reached 32.5 ng/mL. Supplementation with VD_3_ alone, even at a high inclusion level, was insufficient to achieve plasma 25-OHD_3_ concentrations associated with minimal oxidative stress. Collectively, these findings provide clear insight into the biological efficacy of 25-OHD_3_ replacing VD_3_ in nursery diets under conditions where total vitamin D levels are maintained at constant levels. Therefore, dietary inclusion of 25-OHD_3_ can be considered to optimize vitamin D status and antioxidant capacity in nursery pigs.

## Figures and Tables

**Table 1:** Diet formulation and calculated chemical composition ^1^.

Ingredients, %	d 0–14 Postweaning	d 14–28 Postweaning
Corn	34.29	43.39
Soybean meal (48% crude protein)	26.30	31.20
Whey, dried	10.00	7.50
Oats	2.50	2.50
HP300 ^2^	5.00	2.00
Lactose	10.00	5.00
Fish meal	3.00	1.50
Animal plasma	3.00	1.50
Soybean oil	2.40	2.00
Corn starch	1.00	1.00
L-Lysine-HCl	0.11	0.12
DL-Methionine	0.16	0.13
L-Threonine	0.08	0.08
Dicalcium phosphate	0.58	0.50
Limestone	0.98	0.98
Salt	0.25	0.25
Trace mineral mix ^3^	0.15	0.15
Vitamin mix ^4^ (vitamin D free)	0.20	0.20
Total	100.00	100.00
Calculated chemical composition		
Metabolizable energy, kcal/kg	3447	3405
Crude protein, %	24.21	23.17
SID ^5^ lysine, %	1.39	1.28
SID methionine + cysteine, %	0.84	0.77
Total Ca, %	0.80	0.70
Total P, %	0.63	0.57
STTD ^5^ P, %	0.40	0.33

^1^ VD_3_ and 25-OHD_3_ were added to formulate experimental diets according to treatment by replacing an equal amount of corn starch. The vitamin D_3_ product provided 30,000 IU/g. The 25-OHD_3_ product contained 137.75 µg/g; 1 µg of 25-OHD_3_ was considered equivalent to 40 IU of VD_3_. The inclusion rates of each product were as follows: (1) 0.0067% vitamin D_3_ product; (2) 0.0033% vitamin D_3_ product + 0.0181% 25-OHD_3_ product; and (3) 0.0363% 25-OHD_3_ product. ^2^ Hamlet Protein, Findlay, OH. ^3^ The trace mineral premix supplied the following per kilogram of diet: 33 mg of Mn as manganous oxide, 110 mg of Fe as ferrous sulfate, 110 mg of Zn as zinc sulfate, 16.5 mg of Cu as copper sulfate, 0.3 mg of I as Ca iodate, 0.3 mg of Se as sodium selenite. ^4^ The vitamin premix supplied the following per kilogram of diet: 11,000 IU of vitamin A, 99 IU of vitamin E, 4.4 mg of vitamin K, 55 µg of vitamin B_12_, 9.9 mg of riboflavin, 31.9 mg of pantothenic acid, 55 mg of niacin, 0.9 mg of folic acid, 3.9 mg of vitamin B_6_, 3.1 mg of thiamin, and 0.3 mg of biotin, 600 mg of choline chloride. ^5^ SID = standardized ileal digestible; STTD = standardized total tract digestible.

**Table 2:** Postweaning growth performance of pigs fed diets supplemented with 25-hydroxycholecalciferol (25-OHD_3_) in nursery period ^1^.

	Treatment ^2^			SEM	*p*-Value	
Vitamin D_3_, IU/kg:	2000	1000	0			
25-OHD_3_, IU/kg:	0	1000	2000		Treatment	VD vs. 25-OHD ^3^
Body weight, kg						
d 0	5.71	5.74	5.71	0.49	0.58	0.69
d 7	6.58	6.50	6.51	0.56	0.74	0.46
d 14	8.66	8.55	8.58	0.67	0.96	0.79
d 21	11.61	11.88	11.78	0.88	0.93	0.72
d 28	15.90	16.40	16.16	1.12	0.81	0.58
ADG, kg/d						
d 0–7	0.124	0.108	0.115	0.02	0.68	0.44
d 7–14	0.296	0.293	0.295	0.03	1.00	0.95
d 14–21	0.422	0.475	0.458	0.04	0.57	0.33
d 21–28	0.613	0.647	0.626	0.04	0.43	0.30
d 0–14 (Phase I)	0.210	0.200	0.205	0.02	0.94	0.77
d 14–28 (Phase II)	0.517	0.561	0.542	0.03	0.41	0.24
d 0–28 (overall)	0.364	0.381	0.373	0.03	0.83	0.60
ADFI, kg/d						
d 0–7	0.238	0.229	0.227	0.02	0.88	0.63
d 7–14	0.458	0.443	0.437	0.04	0.91	0.69
d 14–21	0.707	0.792	0.782	0.05	0.22	0.09
d 21–28	0.944	1.049	1.021	0.06	0.14	0.06
d 0–14 (Phase I)	0.348	0.336	0.332	0.03	0.88	0.64
d 14–28 (Phase II)	0.826	0.920	0.902	0.05	0.15	0.06
d 0–28 (overall)	0.587	0.628	0.617	0.04	0.54	0.30
G:F						
d 0–7	0.525	0.465	0.499	0.04	0.50	0.34
d 7–14	0.635	0.664	0.675	0.03	0.68	0.41
d 14–21	0.589	0.601	0.583	0.02	0.84	0.90
d 21–28	0.647	0.617	0.615	0.02	0.20	0.08
d 0–14 (Phase I)	0.598	0.596	0.616	0.03	0.86	0.82
d 14–28 (Phase II)	0.624	0.610	0.601	0.01	0.34	0.19
d 0–28 (overall)	0.617	0.606	0.606	0.01	0.82	0.55

SEM, standard error of the means. ^1^ *n* = 5 replicate pens per treatment. ^2^ Treatments: (1) basal diet with 2000 IU/kg vitamin D_3_ supplementation, (2) basal diet with 1000 IU/kg vitamin D_3_ + 1000 IU/kg 25-OHD_3_ supplementation, and (3) basal diet with 2000 IU/kg 25-OHD_3_ supplementation. ^3^ Single degree of freedom contrast (2000 IU/kg vitamin D_3_ supplementation vs. 1000 IU/kg vitamin D_3_ + 1000 IU/kg 25-OHD_3_ and 2000 IU/kg 25-OHD_3_ supplementation) was conducted to evaluate the effect of partially or completely replacing vitamin D_3_ with 25-OHD_3_ while maintaining a total vitamin D level of 2000 IU/kg.

**Table 3:** Plasma 25-hydroxycholecalciferol (25-OHD_3_) concentrations (ng/mL) in pigs fed diets supplemented with 25-OHD_3_ in nursery period ^1^.

	Treatment ^2^			SEM	*p*-Value	
Vitamin D_3_, IU/kg:	2000	1000	0			
25-OHD_3_, IU/kg:	0	1000	2000		Treatment	VD vs. 25-OHD ^3^
d 14 postweaning	13.59 ^c^	35.28 ^b^	48.76 ^a^	2.61	0.01	0.01
d 28 postweaning	16.55 ^b^	41.80 ^a^	50.25 ^a^	1.33	0.01	0.01

SEM, standard error of the means. ^a–c^ Different superscripts within a row mean significantly different (*p* < 0.05). ^1^ *n* = 8 replicates per treatment. ^2^ Treatments: (1) basal diet with 2000 IU/kg vitamin D_3_ supplementation, (2) basal diet with 1000 IU/kg vitamin D_3_ + 1000 IU/kg 25-OHD_3_ supplementation, and (3) basal diet with 2000 IU/kg 25-OHD_3_ supplementation. ^3^ Single degree of freedom contrast (2000 IU/kg vitamin D_3_ supplementation vs. 1000 IU/kg vitamin D_3_ + 1000 IU/kg 25-OHD_3_ and 2000 IU/kg 25-OHD_3_ supplementation) was conducted to evaluate the effect of partially or completely replacing vitamin D_3_ with 25-OHD_3_ while maintaining a total vitamin D level of 2000 IU/kg.

**Table 4:** Plasma immune response parameters in pigs fed diets supplemented with 25-hydroxycholecalciferol (25-OHD_3_) in nursery period ^1^.

	Treatment ^2^			SEM	*p*-Value	
Vitamin D_3_, IU/kg:	2000	1000	0			
25-OHD_3_, IU/kg:	0	1000	2000		Treatment	VD vs. 25-OHD ^3^
Plasma IgG, mg/mL						
d 14 postweaning	4.74	5.19	4.29	0.62	0.42	0.99
d 28 postweaning	2.60	2.60	2.74	0.17	0.76	0.72
Plasma IgA, mg/mL						
d 14 postweaning	0.24	0.23	0.22	0.02	0.69	0.41
d 28 postweaning	0.70	0.72	0.60	0.08	0.60	0.72

SEM, standard error of the means. ^1^ *n* = 8 replicates per treatment. ^2^ Treatments: (1) basal diet with 2000 IU/kg vitamin D_3_ supplementation, (2) basal diet with 1000 IU/kg vitamin D_3_ + 1000 IU/kg 25-OHD_3_ supplementation, and (3) basal diet with 2000 IU/kg 25-OHD_3_ supplementation. ^3^ Single degree of freedom contrast (2000 IU/kg vitamin D_3_ supplementation vs. 1000 IU/kg vitamin D_3_ + 1000 IU/kg 25-OHD_3_ and 2000 IU/kg 25-OHD_3_ supplementation) was conducted to evaluate the effect of partially or completely replacing vitamin D_3_ with 25-OHD_3_ while maintaining a total vitamin D level of 2000 IU/kg.

**Table 5:** Bone mineralization parameters in pigs fed diets supplemented with 25-hydroxycholecalciferol (25-OHD_3_) in nursery period ^1^.

	Treatment ^2^			SEM	*p*-Value	
Vitamin D_3_, IU/kg:	2000	1000	0			
25-OHD_3_, IU/kg:	0	1000	2000		Treatment	VD vs. 25-OHD ^3^
Bone mineral content, g	9.65	9.60	9.02	0.68	0.61	0.58
Bone mineral density, g/cm^3^	0.37	0.36	0.36	0.01	0.91	0.67

SEM, standard error of the means. ^1^ *n* = 6 replicates per treatment. ^2^ Treatments: (1) basal diet with 2000 IU/kg vitamin D_3_ supplementation, (2) basal diet with 1000 IU/kg vitamin D_3_ + 1000 IU/kg 25-OHD_3_ supplementation, and (3) basal diet with 2000 IU/kg 25-OHD_3_ supplementation. ^3^ Single degree of freedom contrast (2000 IU/kg vitamin D_3_ supplementation vs. 1000 IU/kg vitamin D_3_ + 1000 IU/kg 25-OHD_3_ and 2000 IU/kg 25-OHD_3_ supplementation) was conducted to evaluate the effect of partially or completely replacing vitamin D_3_ with 25-OHD_3_ while maintaining a total vitamin D level of 2000 IU/kg.

**Table 6:** Plasma superoxide dismutase activity, total antioxidant capacity, and malondialdehyde level in pigs fed diets supplemented with 25-hydroxycholecalciferol (25-OHD_3_) in nursery period ^1^.

	Treatment ^2^			SEM	*p*-Value	
Vitamin D_3_, IU/kg:	2000	1000	0			
25-OHD_3_, IU/kg:	0	1000	2000		Treatment	VD vs. 25-OHD ^3^
Superoxide dismutase, U/mL						
d 14 postweaning	3.53	3.41	3.53	0.42	0.97	0.91
d 28 postweaning	4.50	3.70	4.39	0.62	0.48	0.47
Total antioxidant capacity, mM trolox equivalents						
d 14 postweaning	5.09	4.93	4.35	0.26	0.11	0.15
d 28 postweaning	4.25	4.02	4.21	0.31	0.67	0.58
Malondialdehyde, µM						
d 14 postweaning	11.75	11.97	10.42	0.52	0.11	0.40
d 28 postweaning	12.15 ^a^	9.62 ^b^	10.02 ^b^	0.69	0.05	0.02

SEM, standard error of the means. ^ab^ Different superscripts within a row means significantly different (*p* < 0.05). ^1^ *n* = 8 replicates per treatment. ^2^ Treatments: (1) basal diet with 2000 IU/kg vitamin D_3_ supplementation, (2) basal diet with 1000 IU/kg vitamin D_3_ + 1000 IU/kg 25-OHD_3_ supplementation, and (3) basal diet with 2000 IU/kg 25-OHD_3_ supplementation. ^3^ Single degree of freedom contrast (2000 IU/kg vitamin D_3_ supplementation vs. 1000 IU/kg vitamin D_3_ + 1000 IU/kg 25-OHD_3_ and 2000 IU/kg 25-OHD_3_ supplementation) was conducted to evaluate the effect of partially or completely replacing vitamin D_3_ with 25-OHD_3_ while maintaining a total vitamin D level of 2000 IU/kg.

## Data Availability

The raw data supporting the conclusions of this article will be made available by the authors on request.

## Abbreviations

The following abbreviations are used in this manuscript:

25-OHD_3_	25-hydroxycholecalciferol
ADFI	Average daily feed intake
ADG	Average daily gain
BMC	Bone mineral content
BMD	Bone mineral density
G:F	Gain to feed ratio
Ig	Immunoglobulin
MDA	Malondialdehyde
SOD	Superoxide dismutase
T-AOC	Total antioxidant capacity
VD_3_	Vitamin D_3_
